# Computational repurposing and preclinical validation of colquhounia root tablets for membranous nephropathy

**DOI:** 10.1002/ctm2.1143

**Published:** 2023-02-28

**Authors:** Xia Mao, Kexin Wang, Yudong Liu, Xiaohui Su, Anguo Wu, Lin Chen, Jiangrui Wang, Beilei Cai, Yanqiong Zhang, Feng Huang, Na Lin

**Affiliations:** ^1^ Research Center of Traditional Chinese Medicine theory and literatures Institute of Chinese Materia Medica China Academy of Chinese Medical Sciences Beijing China; ^2^ Sichuan Key Medical Laboratory of New Drug Discovery and Druggability Evaluation Luzhou Key Laboratory of Activity Screening and Druggability Evaluation for Chinese Materia Medica School of Pharmacy Southwest Medical University Luzhou China; ^3^ Pharmceutical Factory of the Chongqing Academy of Medica Chongqing China; ^4^ Sinomune Pharmaceutical Co. Ltd Wuxi China; ^5^ School of Chinese Materia Medica and Yunnan Key Laboratory of Southern Medicinal Utilization Yunnan University of Chinese Medicine Kunming China

Dear Editor,

Current therapeutics of membranous nephropathy (MN) hold dilemmas of limited clinical response rates, high recurrence rate, and unavoidable adverse effects.^1^ Colquhounia Root Tablet (CRT) is a Chinese patent medicine prepared from the peeled root of *Tripterygium hypoglaucum (Lévl.) Hutch* (SFDA approval number: Z20027411). Emerging clinical evidence has indicated a potential therapeutic effect of CRT on MN.^2^, ^3^ However, the mechanisms of CRT against MN remain unclear.

In this study, 585 putative targets of CRT were predicted hit by chemical compounds identified by UPLC‐Q‐TOF‐MS (Figure S1 and Table S1) and collected from literatures (Table S2).^4^, ^5^ Then, 692 MN‐related genes were collected by searching clinical symptoms and the related genes (Table S3). After constructing the ‘disease‐related genes‐drug putative targets’ interaction network using the links among MN‐related genes and CRT putative targets, a total of 49 key network targets were selected by calculating nodes’ topological features (Table S4). Functionally, the key putative targets of CRT against MN were significantly enriched in 11 signalling pathways, including JAK‐STAT signalling, TNF signalling, PI3K‐Akt signalling and etc. (all *p* < .001, Figure 1A). In the most enriched ‘immunization’ module, tumor necrosis factor α (TNF‐α), interleukin 6 (IL‐6) and their downstream partners (JAK2, STAT3, MMP9) formed a signal axis that may play an essential role in the pathogenesis of MN. Thus, we hypothesized that CRT might alleviate the severity of MN through regulating TNF‐α‐IL‐6‐JAK2/STAT3‐MMP9 signalling axis (Figure 1B,C).

**FIGURE 1:**
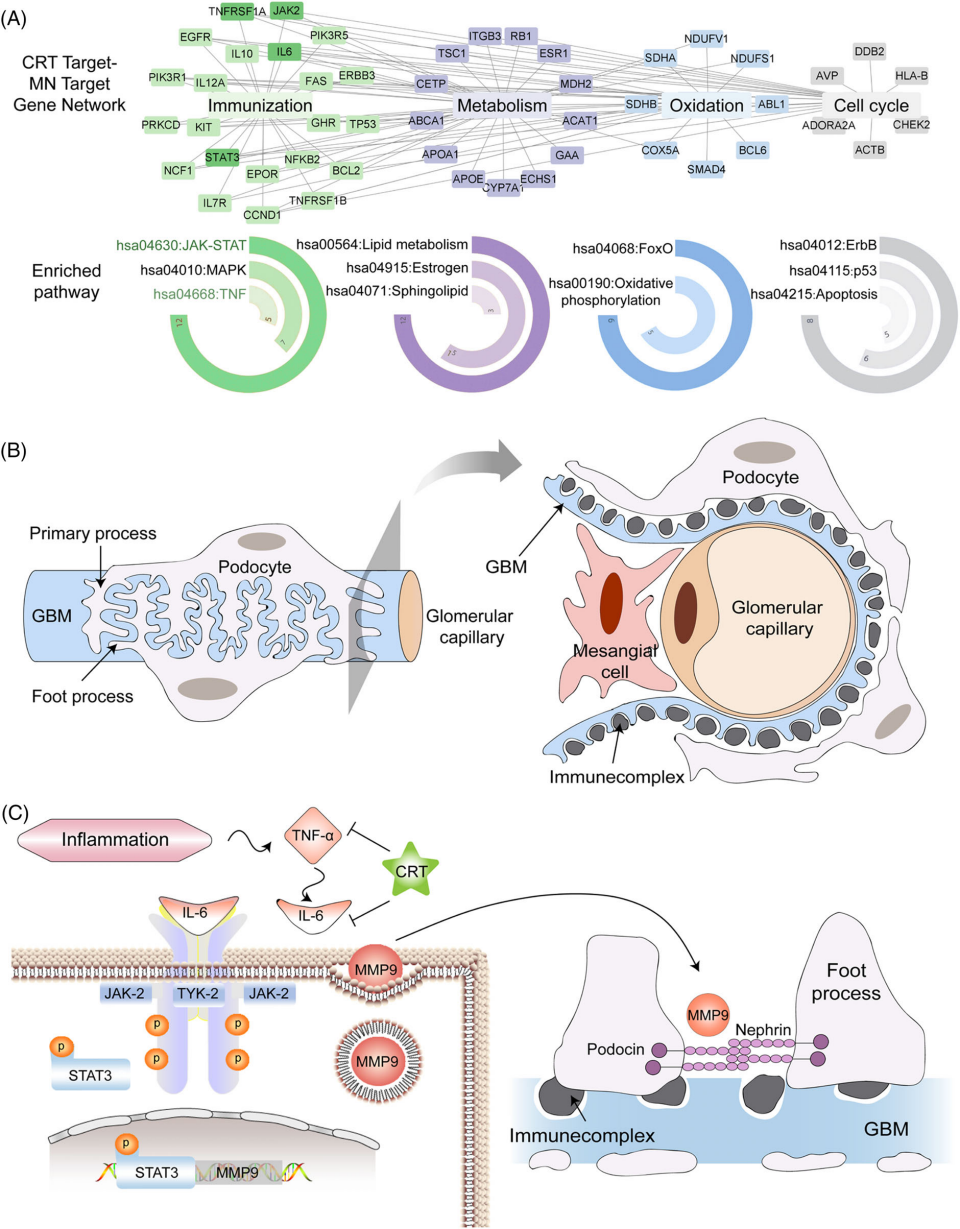
Network‐based investigation of pharmacological mechanisms of Colquhounia Root Tablet (CRT) against membranous nephropathy (MN). (A) Visualization of ‘disease‐related gene‐drug putative target’ interaction network and its functional modules. In the network, the larger rectangles represent the functional modules, the smaller rectangle nodes represent the key network targets of CRT against MN, segment of a circle represent enriched pathways, the longer segment means more node were involved in. (B) The pathological process of MN that characterized with the diffuse thickening of basement membrane, densely accumulation of immune deposits in the subepithelial space, and granuloid‐like deposition of IgG and complement components onto the subepithelial surface of the glomerular capillary wall. (C) Schematic illustration of the TNFα‐IL6‐JAK2/STAT3‐MMP9 signalling axis that may play an essential role in the etiology of MN and in the treatment of CRT

To evaluate the effects of CRT on cationic bovine serum albumin (C‐BSA) induced MN mice, kidney index, levels of microalbumin from 24 h urine, creatinine, total cholesterol and serum albumin were detected after the routine induction of MN mice and drug treatment (Figure 2A). The microalbumin levels from 24 h urine were significantly increased in MN mice from the 4th week till the end of the experiment (*p* < .001, Figure 2B). MN mice showed higher kidney index, higher levels of creatinine and total cholesterol, and lower level of serum albumin than that of normal mice (all *p* < .01), which were all significantly reversed by the treatment of CRT, especially in the CRT‐H group (all *p* < .05), similar to that of prednisone (Figure 2B,F). Immunohistochemical staining showed that MN led to a significant down‐regulation of both nephrin and podocin proteins in MN mice compared with that in the normal group (both *p* < .001). In contrast, the integrity of nephrin and podocin distributions in the glomerular basement membrane (GBM) of MN mice were both rescued effectively at the 8th week following the treatment of CRT and prednisone (both *p* < .01, Figure 2 G,J,K). Pathologically, the GBM and focal segmental mesangial hypercellularity were observed in MN mice using periodic acid‐silver‐meth‐enamine (PASM). Especially, GBM showed a slight thickening, as well as numerous spikes and bubbles (Figure 2H). High dosage of CRT remarkedly improved the morphological changes of renal tissues in MN mice, similar to prednisone (*p* < .001, Figure 2 H,L). An increased granular deposition of IgG along the glomerular capillary walls was also observed in the renal tissues of MN mice compared with normal controls (*p* < .001, Figure 2I,M). MN mice in the CRT and prednisone treatment groups all showed lower levels of IgG deposition than that in the model group (Figure 2I,M). These data suggest that CRT may effectively ameliorate the renal damage and immune injury caused by MN modeling.

**FIGURE 2:**
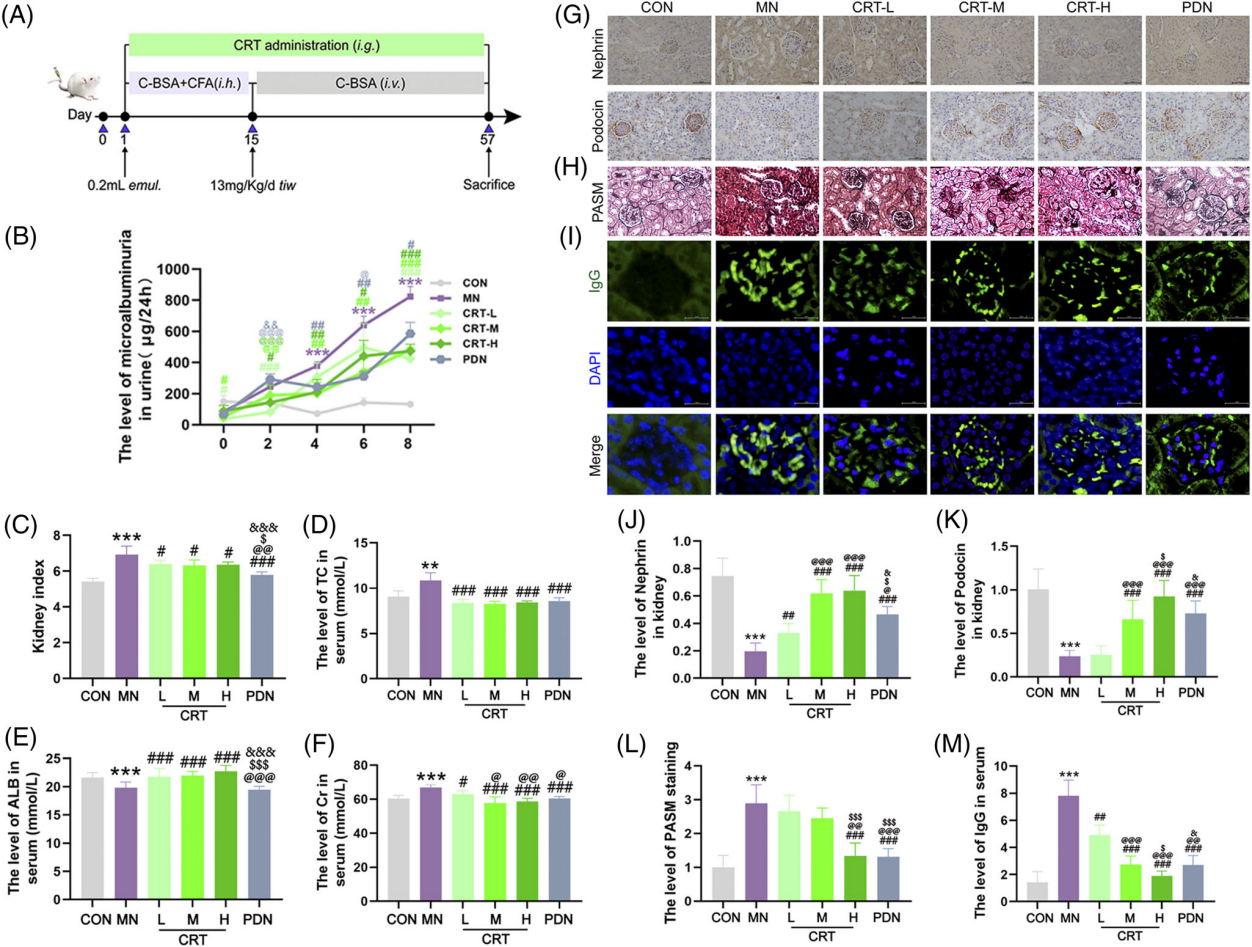
Colquhounia Root Tablet (CRT) effectively reduces the proteinuria and ameliorates the pathological changes in cationic bovine serum albumin (C‐BSA) induced membranous nephropathy (MN) mice. (A) Induction protocol of MN model in female Bagg Albino‐c (BALB/c)mice and drug treatment during the experiments. (B) Levels of microalbumin from 24 h urine of mice in different groups. (C) Kidney index of mice in different groups. (D) Levels of total cholesterol (TC) in mouse serum of different groups. (E) Levels of serum albumin (ALB) of mice in different groups. (F) Levels of serum creatinine (Cr) of mice in different groups. (G) Representative images of nephrin and podocin in renal tissues detected using immunohistochemistry analysis in different groups (400×). (H) Representative images of the glomerulosclerosis in renal tissues detected using periodic acid‐silver‐meth‐enamine (PASM) staining in different groups (400×). (I and M) The co‐localization of images and quantified results of IgG (Green) in renal tissues detected using immunofluorescence analysis. DAPI (Blue) was used to visualize the nuclei, and double‐exposure findings were shown marked Merge (400×). (J and K) Mean intensity results of nephrin and podocin, scored semi quantitatively by Image‐Pro Plus 6.0 software. (L) The semi quantitatively scores of PASM staining in different groups using Image‐Pro Plus 6.0 software. (Normal control group (*n* = 6); MN model group (*n* = 12); CRT‐L treatment group (*n* = 12); CRT‐M treatment group (*n* = 12); CRT‐H treatment group (*n* = 12); Prednisone treatment group (*n* = 12). Experiments were repeated for three times. Data are expressed as the mean ± S.D. ‘^*^’, ‘^**^’, and ‘^***^’, *p* < .05, *p* < .01 and *p* < .001, respectively, comparison with the normal control group. ‘^#^’, ‘^##^’ and ‘^###^’, *p* < .05, *p* < .01 and *p* < .001, respectively, comparison with the MN model group. ‘^@^’, ‘^@@^’ and ‘^@@@^’, *p* < .05, *p* < .01 and *p* < .001, respectively, comparison with the CRT‐L treatment group. ‘^$^’, ‘^$$^’ and ‘^$$$^’, *p* < .05, *p* < .01 and *p* < .001, respectively, comparison with the CRT‐M treatment group. ‘^&^’, ‘^&&^’ and ‘^&&&^, *p* < .05, *p* < .01 and *p* < .001, respectively, comparison with the CRT‐H treatment group

To determine the effects of CRT on podocyte function, mouse podocyte cell line MPC5 was stimulated with C5b‐9, and then treated with CRT or prednisone containing serum (Figure 3A). CCK8 assay demonstrated that the IC50 of CRT against MPC5 cells was 372 µg/ml, providing a reference for dosage design in our in vitro experiments, including CRT‐L (half of IC50, 186 µg/ml), CRT‐M (IC50, 372 µg/ml) and CRT‐H (two times of IC50, 744 µg/ml) groups (Figure 3B). In addition, western blot analysis indicated that C5b‐9 induction markedly down‐regulated the expression levels of nephrin and podocin proteins (both P < .001, Figure 3C,D,E), which was reversed by the treatment of high dosage of CRT more effectively than prednisone (both *p* < .05, Figure 3C,D,E). Notably, the fluorescein isothiocyanate (FITC)‐labelled phalloidin staining of C5b‐9 induced MPC5 cells showed an obvious cell morphology change, with the disorder arrangement of podocyte cytoskeleton, and even the disappearance of normal filamentous structure (*p* < .001, Figure 3G). All dosages of CRT and prednisone treatment effectively ameliorated cytoskeleton destruction of MPC5 cells, to an approximately normal level (all *p* < .01, Figure 3F,G).

**FIGURE 3:**
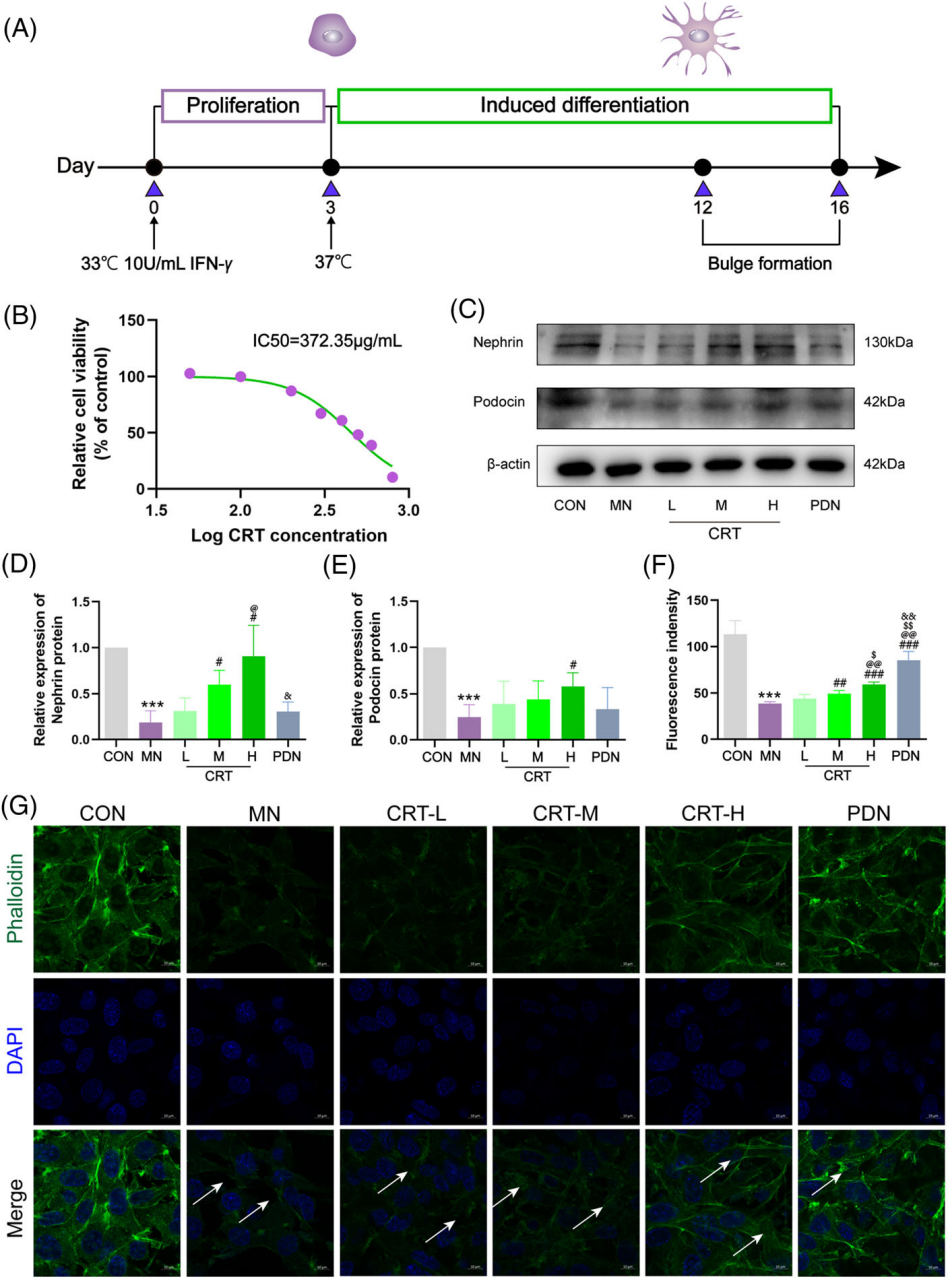
Colquhounia Root Tablet (CRT) protects against podocyte injury based on C5b‐9‐induced MPC5 cells. (A) Induction protocol of MPC5 cells injured by C5b‐9 and drug treatment during the experiments. (B) Cell viability of C5b‐9 induced MPC5 cells in different groups detected using CCK8 assay. (C and E) Expression levels of nephrin and podocin proteins in MPC5 cells of different groups detected using western blot analysis and calculated by Image‐Pro Plus 6.0 software. (F and G) The effects of CRT on the morphology change of C5b‐9 induced MPC5 cells in different groups using fluorescein isothiocyanate (FITC)‐labelled phalloidin (Green) staining. DAPI (Blue) was used to visualize the nuclei, and double‐exposure findings were shown marked Merge (400×). Arrows present podocyte cytoskeleton. Experiments were repeated for three times and sample numbers in each group were three. Data are expressed as the mean ± S.D. ‘^*^’, ‘^**^’ and ‘^***^’, *p* < .05, *p* < .01 and *p* < .001, respectively, comparison with the normal control group. ‘^#^’, ‘^##^’ and ‘^###^’, *p* < .05, *p* < .01 and *p* < .001, respectively, comparison with the membranous nephropathy (MN) model group. ‘^@^’, ‘^@@^’ and ‘^@@@^’, *p* < .05, *p* < .01 and *p* < .001, respectively, comparison with the CRT‐L treatment group. ‘^$^’, ‘^$$^’ and ‘^$$$^’, *p* < .05, *p* < .01 and *p* < .001, respectively, comparison with the CRT‐M treatment group. ‘^&^’, ‘^&&^’ and ‘^&&&^, *p* < .05, *p* < .01 and *p* < .001, respectively, comparison with the CRT‐H treatment group

Mechanically, the expression levels of TNF‐α, IL‐6 and MMP9, as well the ratios of p‐JAK2/JAK2 and p‐STAT3/STAT3 were all significantly increased in the MN mice, compared to the normal mice (all *p* < .01, Figure S2). In contrast, CRT treatment markedly reversed the dysregulation of TNF‐α‐IL‐6‐JAK2/STAT3‐MMP9 signalling axis in MN mice, similar to that of prednisone treatment (all *p* < .05, Figure S2), consistent with the findings of the in vitro experiments (all *p* < .05, Figure 4A‐F). To verify whether TNF‐α‐IL‐6‐JAK2/STAT3‐MMP9 signalling axis might be a target of CRT against podocyte injury, TNF‐α inhibitor infliximab was added to C5b‐induced MPC5 cells. As shown in Figure 4G‐N, infliximab obviously strengthened the regulatory effects of CRT on the corresponding proteins involved in the signalling axis (all *p* < .05). Of note, the modulatory effects of CRT on podocyte injury biomarkers nephrin and podocin were both increased due to the interference of infliximab, compared with that of prednisone (Figure 4K‐I).

**FIGURE 4:**
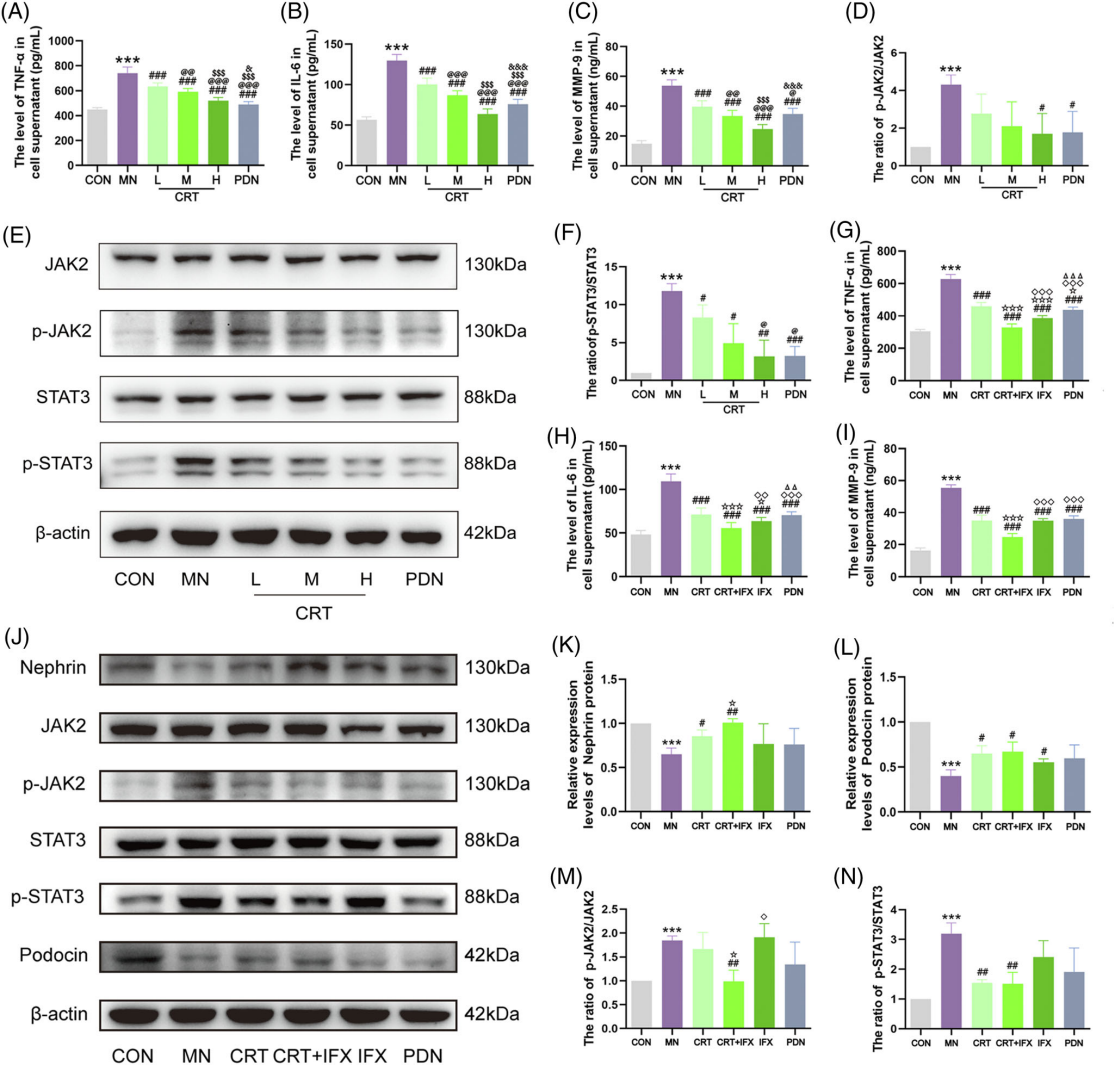
Colquhounia Root Tablet (CRT) effectively alleviate the severity of membranous nephropathy (MN) by targeting TNF‐α‐IL‐6‐JAK2/STAT3‐MMP9 signalling axis based on C5b‐9 induced MPC5 cells. (A–C) Expression levels of TNFα, IL6 and MMP9 proteins in renal tissues of different groups detected using enzyme‐linked immunosorbent assay (ELISA) analysis and calculated by Image‐Pro Plus 6.0 software. (D–F) Ratios of p‐JAK2/JAK2 and p‐STAT3/STAT3 protein expression in renal tissues of different groups detected using western blot analysis and calculated by Image‐Pro Plus 6.0 software. (G–I) Expression levels of TNFα, IL6 and MMP9 proteins in cellular supernatant of different groups detected using ELISA analysis and calculated by Image‐Pro Plus 6.0 software. (J–L) Expression levels of nephrin and podocin proteins in MPC5 cells of different groups detected using western blot analysis and calculated by Image‐Pro Plus 6.0 software. (M and N) Ratios of p‐JAK2/JAK2 and p‐STAT3/STAT3 protein expression in MPC5 cells of different groups detected using western blot analysis and calculated by Image‐Pro Plus 6.0 software. Experiments were repeated for three times. Sample numbers in each group for western blot analysis were three, and that for ELISA analysis were nine. Data are expressed as the mean ± S.D. ‘^*^’, ‘^**^’ and ‘^***^’, *p* < .05, *p* < .01 and *p* < .001, respectively, comparison with the normal control group. ‘^#^’, ‘^##^’ and ‘^###^’, *p* < .05, *p* < .01 and *p* < .001, respectively, comparison with the MN model group. ‘^@^’, ‘^@@^’, and ‘^@@@^’, *p* < .05, *p* < .01 and *p* < .001, respectively, comparison with the CRT‐L treatment group. ‘^$^’, ‘^$$^’ and ‘^$$$^’, *p* < .05, *p* < .01 and *p* < .001, respectively, comparison with the CRT‐M treatment group. ‘^&^’, ‘^&&^’ and ‘^&&&^, *p* < .05, *p* < .01 and *p* < .001, respectively, comparison with the CRT‐H treatment group. ‘^☆^’, ‘^☆☆^’ and ‘^☆☆☆^’, *p* < .05, *p* < .01 and *p* < .001, respectively, comparison with CRT treatment group. ‘^◇^’, ‘^◇◇^’ and ‘^◇◇◇^’, *p* < .05, *p* < .01 and *p* < .001, respectively, comparison with CRT plus infliximab (IFX) treatment group. ‘^Δ^’, ‘^ΔΔ^’ and ‘^ΔΔΔ^’*, p* < .05, *p* < .01 and *p* < .001, respectively, comparison with IFX treatment group

Due to the prominent anti‐inflammation, immune‐regulation and hormone‐like effects,^6^, ^7^ CRT has been authorized by National Medical Products Administration for treating rheumatoid arthritis and systemic lupus erythematosus. Our previous study revealed that CRT effectively ameliorated kidney functions and renal histopathology in diabetic kidney disease through recovering the balance of immune‐inflammation system.^8^ To verify its potentials against MN in a further level, we herein identified chemical and target profilings of CRT and investigated the ‘disease genes‐drug target’ interaction network combined with the in vivo and in vitro experiment validations.

In conclusion, our findings identified CRT as a potential drug candidate for MN and revealed that the treatment of CRT may effectively ameliorate renal dysfunction and reduce the podocyte injury via targeting the TNF‐α‐IL‐6‐JAK2/STAT3‐MMP9 signalling axis, which may facilitate its clinical application in MN therapy.

## CONFLICT OF INTEREST

The authors declare that the research was conducted in the absence of any commercial or financial relationships that could be construed as a potential conflict of interest.

## Supporting information

Supplemantry Information
