# Supplementary material for: Computational repurposing and preclinical validation of colquhounia root tablets for membranous nephropathy
Source: Clin Transl Med. 2023 Feb 28;13(3):e1143. doi: 10.1002/ctm2.1143 (PMC9975455; doi:10.1002/ctm2.1143)
Supplement: Supplementary file 1 — Supplemantry Information [file CTM2-13-e1143-s001.docx]

Supplementary Material

**List of supplementary files:**

**Figure S1. Identification of the main chemical compounds contained in CRT using UPLC-Q-TOF-MS method.**

**Figure S2. CRT effectively inhibits TNFα-IL6-JAK2/STAT3-MMP9 signaling axis in C-BSA induced MN mice.**

**Table S1. Identification of chemical compounds containing in Colquhounia Root Tablet using UPLC-Q-TOF-MS method.**

**Table S2. Chemical information of 43 compounds contained in Colquhounia Root Tablet, that were used for network analysis, obtained via combining the 14 marker compounds identified by UPLC-Q-TOF-MS, and those through literature research in CNKI.**

**Table S3. Membranous nephropathy-associated clinical syndromes used for identifying disease related targets in TCMIP v2.0 platform.**

**Table S4. Topological features of 49 major nodes of Colquhounia Root Tablet against membranous nephropathy (N=49).**

**
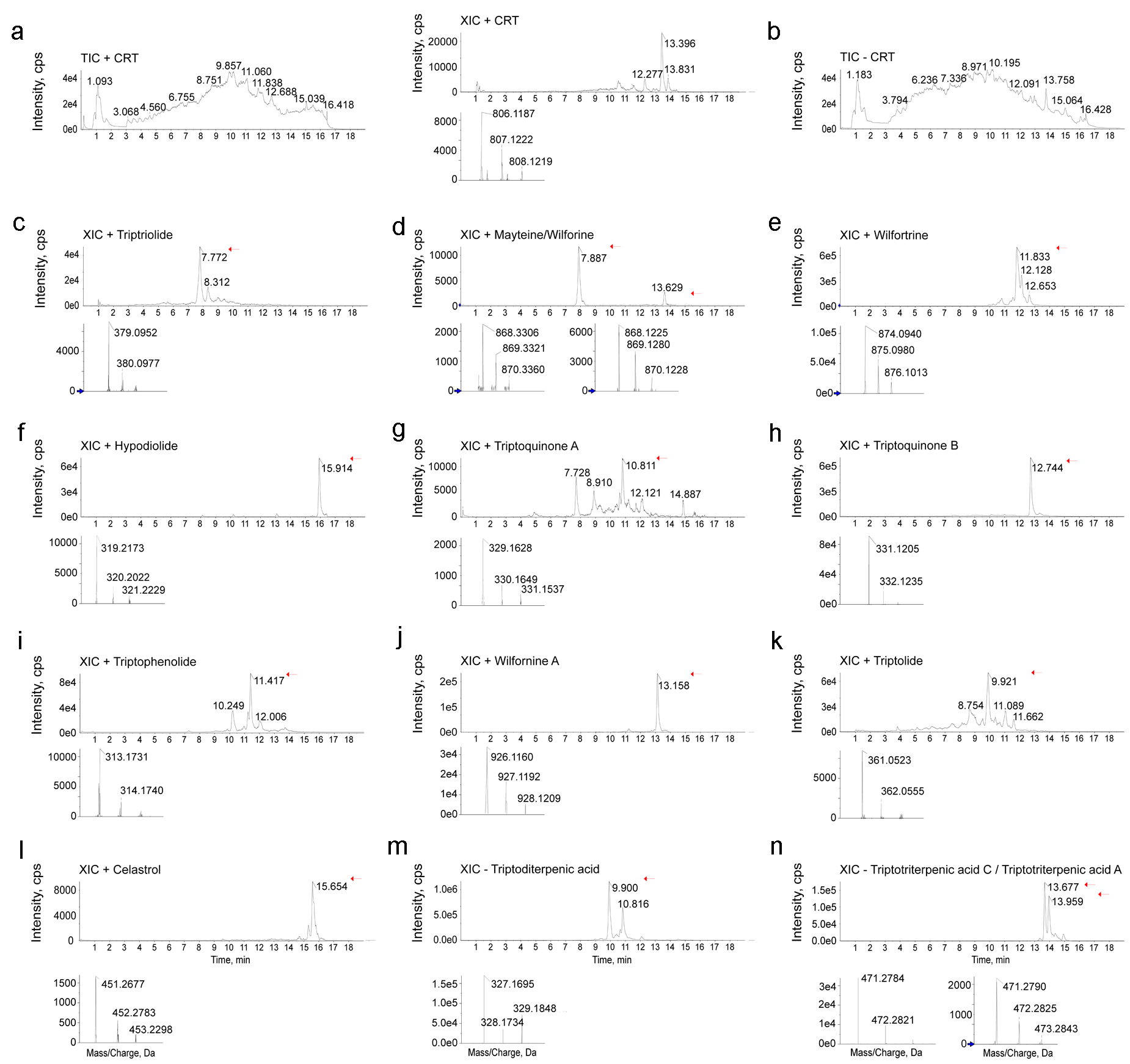
**

**Figure S1. Identification of the main chemical compounds contained in CRT using UPLC-Q-TOF-MS method.** (a~b) CRT. (c) Triptriolide. (d) Mayteine/Wilforine. (e) Wilfortrine. (f) Hypodiolide. (g) Triptoquinone A. (h) Triptoquinone B. (i) Triptophenolide. (j) Wilfornine A. (k) Triptolide. (l) Celastrol. (m) Triptoditerpenic acid. (n) Triptotriterpenic acid A/ Triptotriterpenic acid C.


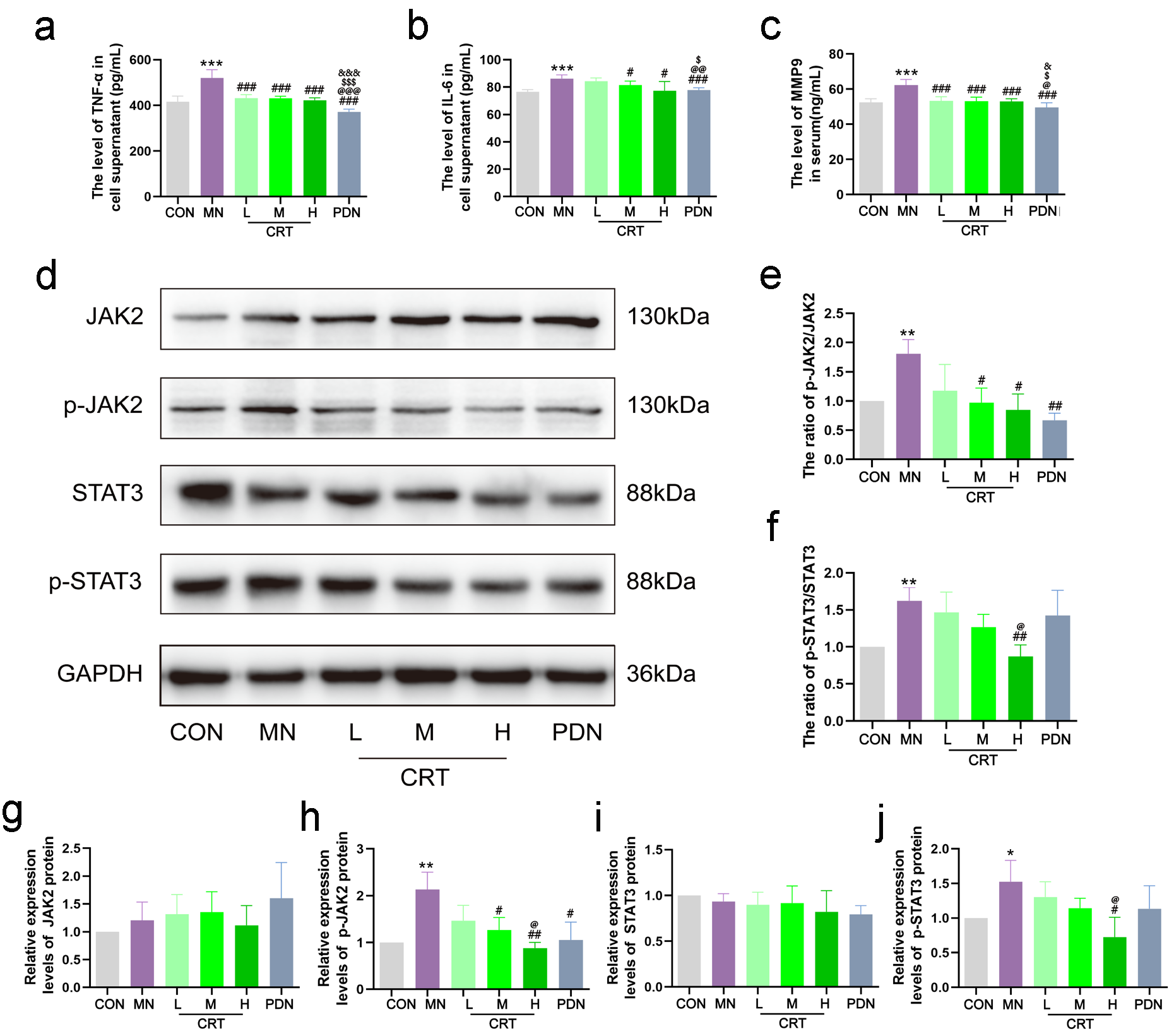


**Figure S2. CRT effectively inhibits TNFα-IL6-JAK2/STAT3-MMP9 signaling axis in C-BSA induced MN mice.** (a~c) Expression levels of TNFα, IL6 and MMP9 proteins in renal tissues of different groups detected using ELISA analysis and calculated by Image-Pro Plus 6.0 software. (d~j) Expression levels of p-JAK2, JAK2, p-STAT3 and STAT3 proteins in renal tissues of different groups detected using western blot analysis and calculated by Image-Pro Plus 6.0 software. Experiments were repeated for three times. Sample numbers in each group for western blot analysis were three, and that for ELISA analysis were five. Data are expressed as the mean±S.D. ‘^*^’, ‘^**^’, and ‘^***^’, P<0.05, P<0.01, and P<0.001, respectively, comparison with the normal control group. ‘^#^’, ‘^##^’, and ‘^###^’, P<0.05, P<0.01, and P<0.001, respectively, comparison with the MN model group. ‘^@^’, ‘^@@^’, and ‘^@@@^’, P<0.05, P<0.01, and P<0.001, respectively, comparison with the CRT-L treatment group. ‘^$^’, ‘^$$^’, and ‘^$$$^’, P<0.05, P<0.01, and P<0.001, respectively, comparison with the CRT-M treatment group. ‘^&^’, ‘^&&^’, and ‘^&&&^, P<0.05, P<0.01, and P<0.001, respectively, comparison with the CRT-H treatment group.

| **Component** | **Formula** | **Molecular Weight** | **Extraction MASS (Da)** | **Adduct** | **Retention time (min)** |
| --- | --- | --- | --- | --- | --- |
| Triptriolide | C_20_H_26_O_7_ | 378.1 | 379.0945 | [M+H]+ | 7.753 |
| Mayteine | C_43_H_49_NO_18_ | 867.8 | 868.3290 | [M+H]+ | 7.562 |
| Wilforine | C_43_H_49_NO_18_ | 867.8 | 868.1187 | [M+H]+ | 13.086 |
| Wilfortrine | C_41_H_47_NO_20_ | 873.8 | 874.0947 | [M+H]+ | 11.847 |
| Hypodiolide | C_20_H_30_O_3_ | 318.4 | 319.2179 | [M+H]+ | 15.918 |
| Triptoquinone A | C_20_H_24_O_4_ | 328.4 | 329.1633 | [M+H]+ | 10.515 |
| Triptoquinone B | C_20_H_26_O_4_ | 330.4 | 331.1205 | [M+H]+ | 12.743 |
| Triptophenolide | C_20_H_24_O_3_ | 312.4 | 313.1717 | [M+H]+ | 11.417 |
| Wilfornine A | C_45_H_51_NO_20_ | 925.9 | 926.1156 | [M+H]+ | 12.838 |
| Triptolide | C_20_H_24_O_6_ | 360.4 | 361.0524 | [M+H]+ | 10.156 |
| Celastrol | C_29_H_38_O_4_ | 450.6 | 451.2690 | [M+H]+ | 15.43 |
| Triptoditerpenic acid | C_21_H_28_O_3_ | 328.4 | 327.1697 | [M-H]- | 9.899 |
| Triptotriterpenic  acid C | C_30_H_48_O_4_ | 472.7 | 471.2784 | [M-H]- | 13.885 |
| Triptotriterpenic  acid A | C_30_H_48_O_4_ | 472.7 | 471.2784 | [M-H]- | 13.885 |

**Table S1. Identification of chemical compounds containing in Colquhounia Root Tablet using UPLC-Q-TOF-MS method**

**Table S2. Chemical information of 43 compounds contained in Colquhounia Root Tablet, that were used for network analysis, obtained via combining the 14 marker compounds identified by UPLC-Q-TOF-MS, and those through literature research in CNKI**

| **Number** | **Compounds** | **CAS** | **Sources** | **Number** | **Compounds** | **CAS** | **Sources** |
| --- | --- | --- | --- | --- | --- | --- | --- |
| 1 | Demethylzeylasteral | 107316-88-1 | CNKI | 23 | Wilforlide A | 84104-71-2 | CNKI |
| 2 | Triptonide | 38647-11-9 | CNKI | 24 | Oleanolic acid | 508-02-1 | CNKI |
| 3 | Triptonolide | 79548-61-1 | CNKI | 25 | Wilforlide B | 84104-70-1 | CNKI |
| 4 | Wilsonine | 39024-12-9 | CNKI | 26 | Procyanidin B2 | 29106-49-8 | CNKI |
| 5 | Triptoquinone | 163513-81-3 | CNKI | 27 | Wilforgine | 37239-47-7 | CNKI |
| 6 | Triptonoterpene | 99694-87-8 | CNKI | 28 | Integracin B | 224186-05-4 | CNKI |
| 7 | Beta-Sitosterol | 83-46-5 | CNKI | 29 | 3-oxo-oleanolic acid | 130216-69-2 | CNKI |
| 8 | Friedelin | 559-74-0 | CNKI | 30 | Triptoquinone A | 142950-86-5 | UPLC-Q-TOF-MS |
| 9 | Emodin | 518-82-1 | CNKI | 31 | Wilfortrine | 37239-48-8 | UPLC-Q-TOF-MS |
| 10 | Neotriptophenolide | 81827-74-9 | CNKI | 32 | Triptoditerpenic acid | 147362-43-4 | UPLC-Q-TOF-MS |
| 11 | Daucosterol | 474-58-8 | CNKI | 33 | Triptotriterpenic acid A | 84108-17-8 | UPLC-Q-TOF-MS |
| 12 | Syringaresinol | 1177-14-6 | CNKI | 34 | Triptotriterpenic acid C | 123914-32-9 | UPLC-Q-TOF-MS |
| 13 | Triptonoterpenol | 110187-23-0 | CNKI | 35 | Celastrol | 34157-83-0 | UPLC-Q-TOF-MS |
| 14 | 3-O-Acetyloleanolic acid | 4339-72-4 | CNKI | 36 | Triptoquinone B | 142937-50-6 | UPLC-Q-TOF-MS |
| 15 | Triptolidenol | 99694-86-7 | CNKI | 37 | Hypodiolide | 139122-81-9 | UPLC-Q-TOF-MS |
| 16 | 16-Hydroxytriptolide | 139713-80-7 | CNKI | 38 | Triptriolide | 137131-18-1 | UPLC-Q-TOF-MS |
| 17 | Tripdiolide | 38647-10-8 | CNKI | 39 | Triptolide | 38748-32-2 | UPLC-Q-TOF-MS |
| 18 | （+）-catechin | 154-23-4 | CNKI | 40 | Mayteine | 104736-05-2 | UPLC-Q-TOF-MS |
| 19 | Wilfordine | 37239-51-3 | CNKI | 41 | Wilfornine A | 345954-00-9 | UPLC-Q-TOF-MS |
| 20 | (-)-Gallocatechin | 3371-27-5 | CNKI | 42 | Wilforine | 11088-09-8 | UPLC-Q-TOF-MS |
| 21 | Triptonoditerpenic acid | 139953-20-1 | CNKI | 43 | Triptophenolide | 74285-86-2 | UPLC-Q-TOF-MS |
| 22 | Beta-Sitosteryl palmitate | 2308-85-2 | CNKI |  |  |  |  |

**Table S3. Membranous nephropathy-associated clinical syndromes used for identifying disease related targets in TCMIP v2.0 platform**

| **Number** | **Disease-related clinical syndromes** | **Number** | **Disease-related clinical syndromes** |
| --- | --- | --- | --- |
| 1 | Abnormality of lipid metabolism | 11 | Hypertriglyceridemia |
| 2 | Albuminuria | 12 | Hypoalbuminemia |
| 3 | Azotemia | 13 | Hypoproteinemia |
| 4 | Congenital nephrotic syndrome | 14 | Increased blood urea nitrogen |
| 5 | Decreased glomerular filtration rate | 15 | Microalbuminuria |
| 6 | Elevated serum creatinine | 16 | Microscopic hematuria |
| 7 | Generalized edema | 17 | Oliguria |
| 8 | Hematuria | 18 | Renal insufficiency |
| 9 | Hypercholesterolemia | 19 | Thickening of the glomerular basement membrane |
| 10 | Hyperlipidemia |  |  |

**Table S4. Topological features of 49 major nodes of Colquhounia Root Tablet against membranous nephropathy**

| **Number** | **Node** | **Degree** | **Closeness Centrality** | **Betweenness Centrality** |
| --- | --- | --- | --- | --- |
| 1 | PIK3R1 | 37 | 0.048 | 11647.65 |
| 2 | TP53 | 31 | 0.048 | 7530.97 |
| 3 | EGFR | 30 | 0.048 | 8394.968 |
| 4 | TNFRSF1A | 27 | 0.048 | 5604.862 |
| 5 | NFKB2 | 27 | 0.047 | 3864.144 |
| 6 | TNFRSF1B | 27 | 0.047 | 3795.542 |
| 7 | CHEK2 | 26 | 0.048 | 9734.142 |
| 8 | ABL1 | 25 | 0.048 | 5031.709 |
| 9 | JAK2 | 24 | 0.048 | 4113.864 |
| 10 | MDH2 | 24 | 0.047 | 3478.246 |
| 11 | PRKCD | 23 | 0.048 | 6291.557 |
| 12 | STAT3 | 23 | 0.048 | 4561.734 |
| 13 | APOA1 | 23 | 0.047 | 3883.979 |
| 14 | NDUFS1 | 23 | 0.047 | 1568.451 |
| 15 | NDUFV1 | 23 | 0.047 | 1528.085 |
| 16 | BCL2 | 21 | 0.048 | 4353.858 |
| 17 | ACTB | 21 | 0.047 | 3332.51 |
| 18 | TSC1 | 21 | 0.048 | 3026.297 |
| 19 | IL6 | 21 | 0.047 | 2479.889 |
| 20 | HLA-B | 19 | 0.047 | 5346.172 |
| 21 | SMAD4 | 19 | 0.047 | 3856.637 |
| 22 | ACAT1 | 19 | 0.047 | 3218.69 |
| 23 | RB1 | 19 | 0.048 | 3134.329 |
| 24 | IL7R | 19 | 0.047 | 2575.229 |
| 25 | ITGB3 | 19 | 0.047 | 2539.038 |
| 26 | ECHS1 | 19 | 0.048 | 2467.918 |
| 27 | IL10 | 19 | 0.047 | 1868.31 |
| 28 | EPOR | 19 | 0.047 | 1769.243 |
| 29 | GHR | 18 | 0.047 | 3163.3 |
| 30 | GAA | 18 | 0.047 | 2929.772 |
| 31 | SDHA | 18 | 0.047 | 2409.544 |
| 32 | COX5A | 18 | 0.047 | 2033.403 |
| 33 | KIT | 18 | 0.047 | 2011.223 |
| 34 | FAS | 18 | 0.048 | 1934.411 |
| 35 | PIK3R5 | 18 | 0.047 | 1566.487 |
| 36 | ERBB3 | 18 | 0.047 | 1549.598 |
| 37 | CYP7A1 | 18 | 0.047 | 1486.179 |
| 38 | ABCA1 | 18 | 0.047 | 1485.711 |
| 39 | CCND1 | 18 | 0.047 | 1426.809 |
| 40 | CETP | 18 | 0.047 | 1398.786 |
| 41 | ESR1 | 17 | 0.047 | 1982.042 |
| 42 | ADORA2A | 17 | 0.047 | 1951.408 |
| 43 | BCL6 | 17 | 0.047 | 1793.025 |
| 44 | NCF1 | 17 | 0.047 | 1734.709 |
| 45 | APOE | 17 | 0.047 | 1596.974 |
| 46 | IL12A | 17 | 0.047 | 1412.788 |
| 47 | SDHB | 17 | 0.047 | 1412.132 |
| 48 | AVP | 17 | 0.047 | 1396.929 |
| 49 | DDB2 | 17 | 0.047 | 1396.481 |
